# Hypoxia/Reoxygenation of Rat Renal Arteries Impairs Vasorelaxation via Modulation of Endothelium-Independent sGC/cGMP/PKG Signaling

**DOI:** 10.3389/fphys.2018.00480

**Published:** 2018-05-03

**Authors:** Diana Braun, Christa Zollbrecht, Stefanie Dietze, Rudolf Schubert, Stefan Golz, Holger Summer, Pontus B. Persson, Mattias Carlström, Marion Ludwig, Andreas Patzak

**Affiliations:** ^1^Renal Vessel Physiology Group, Institute of Vegetative Physiology, Charité – Universitätsmedizin Berlin, Berlin, Germany; ^2^Institute of Vegetative Physiology, Charité – Universitätsmedizin Berlin, Berlin, Germany; ^3^Department of Physiology and Pharmacology, Karolinska Institutet, Stockholm, Sweden; ^4^Centre for Biomedicine and Medical Technology Mannheim, Research Division Cardiovascular Physiology, Medical Faculty Mannheim, Heidelberg University, Mannheim, Germany; ^5^Bayer Pharma AG, Wuppertal, Germany

**Keywords:** hypoxia, ischemia/reperfusion injury, nitric oxide, cGMP, sGC, vascular smooth muscle, relaxation

## Abstract

Ischemia/reperfusion injury holds a key position in many pathological conditions such as acute kidney injury and in the transition to chronic stages of renal damage. We hypothesized that besides a reported disproportional activation of vasoconstrictor response, hypoxia/reoxygenation (H/R) adversely affects endothelial dilatory systems and impairs relaxation in renal arteries. Rat renal interlobar arteries were studied under isometric conditions. Hypoxia was induced by application of 95% N_2_, 5% CO_2_ for 60 min to the bath solution, followed by a 10 min period of reoxygenation (95% O_2_, 5% CO_2_). The effect of H/R on relaxation was assessed using various inhibitors of endothelial dilatory systems. mRNA expression of phosphodiesterase 5 (PDE5), NADPH oxidases (NOX), and nitric oxide synthase (NOS) isoforms were determined using qRT-PCR; cGMP was assayed with direct cGMP ELISA. Acetylcholine induced relaxation was impaired after H/R. Inhibition of the NOS isoforms with L-NAME, and cyclooxygenases (COXs) by indomethacin did not abolish the H/R effect. Moreover, blocking the calcium activated potassium channels K_Ca3.1_ and K_Ca2.1_, the main mediators of the endothelium-derived hyperpolarizing factor, with TRAM34 and UCL1684, respectively, showed similar effects in H/R and control. Arterial stiffness did not differ comparing H/R with controls, indicating no impact of H/R on passive vessel properties. Moreover, superoxide was not responsible for the observed H/R effect. Remarkably, H/R attenuated the endothelium-independent relaxation by sodium nitroprusside, suggesting endothelium-independent mechanisms of H/R action. Investigating the signaling downstream of NO revealed significantly decreased cGMP and impaired relaxation during PDE5 inhibition with sildenafil after H/R. Inhibition of PKG, the target of cGMP, did not normalize SNP-induced relaxation following H/R. However, the soluble guanylyl cyclase (sGC) inhibitor ODQ abolished the H/R effect on relaxation. The mRNA expressions of the endothelial and the inducible NOS were reduced. NOX and PDE5 mRNA were similarly expressed in H/R and control. Our results provide new evidence that impaired renal artery relaxation after H/R is due to a dysregulation of sGC leading to decreased cGMP levels. The presented mechanism might contribute to an insufficient renal reperfusion after ischemia and should be considered in its pathophysiology.

## Introduction

Its function and anatomical structure render the kidney very susceptible to ischemia/reperfusion injury (IRI). IRI has been reported in renal transplantation and, amongst others, in the development of acute kidney injury (AKI), diabetic nephropathy and contrast agent-induced nephropathy (Reuter and Mrowka, 2015; Maringer and Sims-Lucas, 2016). IRI may result from insufficient renal perfusion of different origin, followed by the re-establishment of blood flow (Le Dorze et al., 2009). In contrast to many other organs, where ischemia is followed by an exaggerated flow in the reperfusion period, renal blood flow re-establishes slowly over a period of many minutes (Schneider et al., 2010; Cantow et al., 2017). Moreover, no-reflow phenomenon has been described in IRI experiments (Patschan et al., 2012). This delayed restoration of renal blood flow after ischemia may reflect kidney specific blood vessel properties and/or interactions of renal vasculature with the surrounding tubules. In addition, renal vasculature may be affected by the activation of the sympathetic nervous system and the renin–angiotensin system (RAS) resulting in an increased vascular resistance in IRI (Kontogiannis and Burns, 1998; Mutoh et al., 2014). Experiments in isolated renal arteries of mice revealed increased reactivity to angiotensin II (Ang II) after hypoxia/reoxygenation (H/R) in the presence of norepinephrine (NE) in physiological concentrations, supporting the idea of a concerted action of both systems on renal vascular tone. H/R may modify vessel function by several mechanisms. Besides the proposed disproportional activation of vasoconstrictor systems and a potential increase in oxidative stress including enhanced superoxide levels, endothelial dilatory systems could be equally affected by H/R (Jankauskas et al., 2016; Pahlitzsch et al., 2018). However, no data exist regarding the direct influence of H/R on renal vessel relaxation. Endothelial-derived vasoactive substances, such as nitric oxide (NO), cyclooxygenase (COX)-synthesized prostaglandins or the endothelial-derived hyperpolarizing factor (EDHF) might be responsible for the vasodilatory response following H/R. The gasotransmitter NO is a potent vasodilator and exerts its vasodilatory effects via different pathways. Endogenously produced NO diffuses to vascular smooth muscle cells (VSMCs), where it initiates relaxation mainly via the soluble guanylyl cyclase/cyclic GMP/PKG (sGC/cGMP/PKG) pathway (Denninger and Marletta, 1999). H/R might compromise any step of this pathway leading to impaired relaxation. Interestingly, the effect of H/R on the balance of vasoconstriction and relaxation is sparsely investigated in the pathophysiology of IRI. In the present study, we aimed at investigating the vascular effects of H/R in the presence of physiological NE concentrations in a rat model of renal arterial vessels [interlobar arteries (ILAs)] and studied endothelium-dependent and independent relaxation. Results indicate that H/R impairs relaxation via endothelium-independent mechanisms, adversely affecting sGC/cGMP signaling. We therefore propose that unbalanced vasodilator systems in contrast to enhanced vasoconstriction should be reassessed concerning the pathophysiology of IRI and considered in future studies of the renal vasculature.

## Materials and Methods

### Experimental Animals

Male Sprague-Dawley rats (purchased from Charles River, Sulzfeld, Germany) (270–400 g, 10 weeks of age, *n* = 140) were included in the study. The animals were given food and water *ad libitum*. They were housed in the animal house of the Charité – Universitätsmedizin Berlin at a controlled ambient temperature of 22°C with 50% ± 10% relative humidity and with a 12-h light/12-h dark cycle. Protocols and animal handling were in accordance with the NIH Guide for the Care and Use of Laboratory Animals. Experiments were approved by the Office for Health and Social Matters of Berlin (Berlin, Germany, T0061/08).

### Chemicals

The following drugs were used: acetylcholine (ACh), phenylephrine (PE), sodium nitroprusside (SNP), NE, L-NAME (Nω-nitro-L-arginine methylester hydrochloride), indomethacin, TRAM34 (1-[(2-chlorophenyl)diphenylmethyl]-1H-pyrazole), UCL 1684 (6,12,19,20,25,26-hexahydro-5,27:13,18:21,24-trietheno-11,7-metheno-7H-dibenzo [b,n] [1,5,12,16] tetraazacyclotricosine-5,13-diium dibromide), sildenafil citrate, 4-hydroxy-TEMPO, 8-bromoguanosine 3′,5′-cyclic monophosphate sodium salt (8-Br-cGMP) ODQ (1H-[1,2,4]oxadiazolo[4,3-a]quinoxalin-1-one) and DT-2 trifluoroacetate salt (DT-2). All substances were obtained from Sigma-Aldrich (Darmstadt, Germany) with the exception of indomethacin, ODQ (Cayman Chemical Company, Ann Arbor, MI, United States) and sildenafil citrate (Biomol GmbH, Hamburg, Germany). Distillated water was used to prepare the chemical solutions, except for indomethacin, TRAM34, UCL 1684, ODQ and sildenafil citrate, which were dissolved in >99.7% dimethyl sulfoxide (DMSO). The final concentration of DMSO was below 0.1% in all of the experiments. SNP was dissolved in distillated water immediately before start of the experiment in lightproof tubes. Concentrations are given as final molar concentration in the bath solution.

### Preparation of Rat Interlobar Arteries

The animals were killed by decapitation under isoflurane. The kidneys were removed immediately and placed in cold preparation solution (146 mmol/NaCl, 4.5 mmol/l KCl, 1.2 mmol/l NaH_2_PO_4_^∗^2H_2_O, 1 mmol/l MgSO_4_^∗^7H_2_O, 5.5 mmol/l glucose, 0.025 mmol/l Na (EDTA), 5 mmol/l HEPES, and 0.1 mmol/l CaCl_2_^∗^2H_2_O; pH 7.4). Kidneys were sliced along the main axis of tubular and vascular structures. ILA were dissected and arterial sections (length: 2 mm) were isolated.

### Measurements of Vascular Responses

Interlobar arteries were mounted on 40-μm stainless steel wires in a small vessel myograph (model 410A, DMT, Denmark) filled with cold preparation solution. After mounting, the solution was changed to experimental solution (119 mmol NaCl, 4.7 mmol/l KCl, 1.2 mmol/l KH_2_PO_4_, 1.2 mmol/l MgSO_4_^∗^7H_2_O, 6.1 mmol/l glucose, 25 mmol/l NaHCO_3_, and 2.5 mmol/l CaCl_2_^∗^2H_2_O; pH 7.4). The myograph was heated up to 37°C under unlimited gas flow of carbogen (95% O_2_, 5% CO_2_). Thereafter, vessels underwent a normalization procedure to an internal circumference equivalent to 90% of that produced under an intramural pressure of 100 mmHg. The viability of ILA was demonstrated by application of K-PSS (123.7 mmol/l KCl, 1.2 mmol/l KH_2_PO_4_, 1.2 mmol/l MgSO_4_^∗^7H_2_O, 6.1 mmol/l glucose, 25 mmol/l NaHCO_3_ and 2.5 mmol/l CaCl_2_^∗^2H_2_O; pH 7.4). Endothelial function was tested by cumulative concentration–response relationships to ACh (10^-9^ to 10^-5^ mol/l) on arteries precontracted with PE to 50–70% of the maximum arterial force to K-PSS. Only arteries showing complete contraction to K-PSS and relaxation (>70%) to ACh were used for the subsequent experiments. In initial experiments, the effect of pO_2_ on vasoreactivity was tested and ILA exposed to either ambient O_2_ (21% O_2_) or carbogen (95% O_2_). For all following experiments, ILA were exposed to hypoxic gas (95% N_2_, 5% CO_2_) or carbogen (control) for 60 min followed by 10 min of reoxygenation with carbogen. NE in a non-pressor concentration (10^-9^ mol/l) was applied during the hypoxic and the control situation to simulate a sympathetic activity. NE was washed out during the reoxygenation period. Data were digitized using PowerLab system (ADinstruments, Spechbach, Germany).

### Measurements of Endothelial Dilatory Function

**FIGURE 1:**
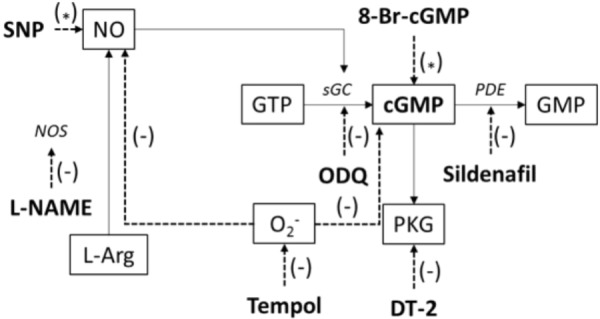
Illustration of applied chemicals (bold) to affect the NO/sGC/cGMP signaling pathway; SNP, sodium nitroprusside; NO, nitric oxide; NOS, nitric oxide synthase; L-NAME, Nω-nitro-L-arginine methylester hydrochloride; L-Arg, L-arginine; O_2_^-^, superoxide; GTP, guanosine triphosphate; sGC, soluble guanylyl cyclase; ODQ, 1H-[1,2,4]oxadiazolo[4,3-a]quinoxalin-1-one; GMP, guanosine monophosphate; cGMP, cyclic guanosine monophosphate; 8-Br-cGMP, 8-bromoguanosine 3’,5’-cyclic monophosphate sodium salt; PKG, cGMP-dependent protein kinase; DT-2, DT-2 trifluoroacetate salt; PDE, phosphodiesterase; tempol, 4-hydroxy-TEMPO. (^∗^) – activation, (–) – inhibition.

**Figure 1** represents a scheme to illustrate the protocols used to investigate the endothelium-independent pathways and to affect the NO/sGC/cGMP signaling.

L-NAME [inhibitor of endothelial NO synthase (eNOS), inducible NOS (iNOS), and neuronal NOS (nNOS), 10^-4^ mol/l], or indomethacin (inhibitor of COXs, 10^-5^ mol/l), or TRAM34 (K_Ca_3.1 blocker, 10^-6^ mol/l), UCL 1684 (K_Ca_2.3 blocker, 10^-7^ mol/l) or ODQ (sGC inhibitor, 10^-5^ mol/l) were applied during the entire 60 + 10 min period. Immediately after the reoxygenation period, ACh concentration–response relationships (10^-9^ to 10^-5^ mol/l) were obtained from PE precontracted ILA in the presence or absence of the mentioned drugs.

### Measurements of Endothelium-Independent Relaxation

Sodium nitroprusside was used for endothelium-independent induction of relaxation. Concentration–response relationships to SNP (10^-9^ to 10^-4^ mol/l) were obtained from precontracted ILA (PE), while the endothelial dilatory systems via NOS and COX were inhibited by L-NAME (10^-4^ mol/l) and indomethacin (10^-5^ mol/l), respectively. To test the role of cGMP as an important mediator of NO induced relaxation, 8-bromoguanosine 3′,5′-cyclic monophosphate sodium salt (10^-8^ to 3 × 10^-4^ mol/l), a stable form of cGMP, was added to precontracted ILA. The downstream target PKG was investigated by inhibition using DT-2 trifluoroacetate salt (10^-7^ mol/l). Further, a potential role of PDE5 for the H/R effect on relaxation was tested by applying sildenafil citrate (10^-8^ to 10^-5^ mol/l). To exclude an influence of oxidative stress, 4-hydroxy-TEMPO (tempol, 10^-4^ mol/l) was added during the 60 + 10 min H/R period.

### Measurements of Vascular Wall Stiffness

Length-tension relationships were obtained by stretching the vessels in 20 μm steps until the wall tension was 4.5 mN/mm after the 60 + 10 min H/R period in order to investigate mechanical properties of the VSMCs in ILA. The experimental data of each vessel were fitted by using the equation T = T_0_ exp [beta^∗^(IC-IC_0_)/IC_0_]. T is the wall tension (mN/mm), T_0_ is the wall tension at the transmural pressure of 100 mmHg, IC_0_ is the internal circumference (mm) of the vessels at the transmural pressure of 100 mmHg and IC is the internal circumference (mm) of the vessels. The parameter beta expresses vascular wall stiffness (Freslon and Giudicelli, 1983; Nyborg et al., 1987).

### Preparation of Rat Interlobar Arteries for Gene Expression Analysis

Isolated ILA were kept in experimental solution at 37°C in an oxygenation chamber and were exposed to hypoxic gas or carbogen for 60 min followed by 10 min of reoxygenation with carbogen. NE (10^-9^ mol/l) was applied during the hypoxic and control period and was washed out during reoxygenation.

### RNA Isolation and Quantitative PCR

The mRNA was promptly isolated. The tissue was homogenized in lysis buffer (1.0% NP40, 0.5% sodium deoxycholate, 0.1% SDS, 10 mmol/l NaF, 80 mmol/l Tris, pH 7.5) containing enzyme inhibitors (Complete Mini; 1 tablet/1.5 ml; Roche Diagnostics, Mannheim, Germany). RNA was isolated with RNA-Bee-reagent (Biozol, Eching, Germany) and reverse transcribed with random hexamers (High Capacity cDNA RT-Kit, Applied Biosystems, Foster City, CA, United States, #4374966), according to the manufacturer’s protocols. QPCR was performed using a Lightcycler LC480 (Roche Diagnostics, Mannheim, Germany), according to the manufacturer’s protocols and with a StepOnePlus device (Applied Biosystems, Foster City, CA, United States) using SYBR Green for fluorescent detection of DNA generated during PCR, respectively. The expression levels of mRNA were normalized to 18SrRNA and the housekeeping gene RPL32, respectively (primer sequences: see **Table 1**). Relative expressions to control situation were calculated by using the 2^-ΔΔ^Ct-method.

**Table 1:** Primer for the mRNA expression studies.

Primer	Forward	Reverse
nNOS	ATCCAGGTGGACAGAGACCTCGATG	CCGAGGTAGGGGACTGTTCCTTCTCT
iNOS	CAGGTGCTATTCCCAGCCCAACA	CATTCTGTGCAGTCCCAGTGAGGAA
eNOS	CAGCACCAGACCACAGCCCC	TCCTGCTGAGCCTGTGCACT
NOX1	CGTCACTCCCTTTGCTTCCT	AGGCACCCGTCTCTCTACAA
NOX2	CTGCCAGTGTGTCGGAATCT	ACACACCACTCCACGTTGAA
NOX4	TTGGTGAACGCCCTGAACTT	TACCACCACCATGCAGACAC
18S rRNA	ACGGACCAGAGCGAAAGCAT	TGTCAATCCTGTCCGTGTCC
PDE5A	GTCTGCCCAAACCCTTAAAA	CGCTGTTTCCAGATCAGACA
RPL32	GAAAGAGCAGCACAGCTGGC	TCATTCTCTTCGCTGCGTAGC

### Analysis of Reactive Oxygen Species Formation and Distribution

Interlobar arteries were mounted and exposed to hypoxia or carbogen for 60 min in the presence of NE (10^-9^ mol/l), followed by a reoxygenation step of 10 min. During the treatment ILA were incubated with dihydroethidium (DHE) (2^∗^10^-5^ mol/l) for 30 min followed by wash-out. ILA were immediately frozen in tissue tek (Sakura, Staufen, Germany). Cryosections (10 μm) were counterstained with DAPI (4′,6-diamidino-2-phenylindole, dihydrochloride). DHE fluorescence was captured using a Zeiss Axiophot fluorescence microscope (Zeiss Axiovert 200M, software Axiovision 3.0). For each vessel, DHE fluorescence was measured in four separate cryosections (four arterial rings) and calculated from 10 separate high power fields/image using ImageJ software (NIH, Bethesda, MD, United States).

### Direct cGMP ELISA

Interlobar arteries were isolated (see gene expression analysis) and exposed to hypoxia or carbogen for 60 min in the presence of NE (10^-9^ mol/l), followed by a reoxygenation step of 10 min. ILA were frozen and stored at -80°C until assay. The cGMP levels in ILA were measured using a direct cGMP ELISA kit (Enzo Life Sciences, Lausen, Switzerland) according to the manufacturer’s instruction.

### Statistical Analyses

EC_50_ were calculated by fitting the concentration–response relationship using a non-linear four parameter logistic function. Differences in EC_50_ were tested by a non-parametric Wilcoxon test for independent variables (Sigmaplot 13.0, Systat Software GmbH, Erkrath, Germany). If applicable, differences during the concentration–response relationships were tested by ANOVA for repeated measurements and non-normal distribution (Brunner test, “R” project^1^). ANOVA followed by Wilcoxon test and correction for multiple comparisons were used for testing group differences of expression. mRNA expression data were tested by the Mann–Whitney test. Data are presented as mean ± SEM. The *n*-value given in the figures corresponds to vessel number. The confidence level *P* was set to 0.05.

## Results

### Impaired Relaxation of Rat Interlobar Arteries After H/R

**FIGURE 2:**
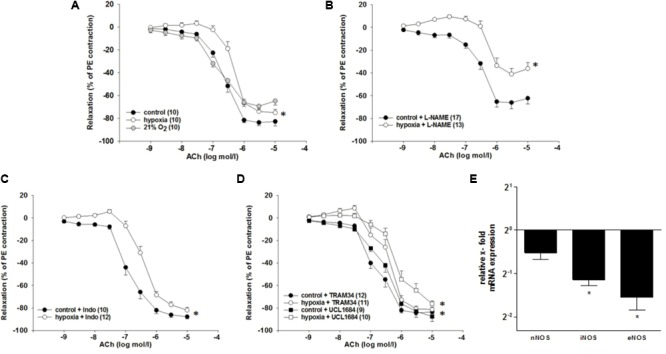
Effects of H/R and inhibitors of dilatory systems on endothelium-dependent relaxation in isolated rat renal interlobar arteries (ILAs) precontracted with PE. **(A)** Carbogen (control) showed no adverse effects on relaxation compared to 21% O_2_. EC_50_ of H/R compared to control was significantly different pointing to diminished ACh-induced relaxation following H/R [EC_50_: 2.37^∗^10^-7^ mol/l (control) vs. 4.75^∗^10^-7^mol/l (H/R); *P* < 0.05]. Inhibiting endothelial dilatory systems **(B–D)** did not reverse the effect of H/R on ILA relaxation. **(B)** NOS inhibition [with L-NAME, EC_50_: 3.33^∗^10^-7^ mol/l (control) vs. 9.54^∗^10^-7^ mol/l (H/R); *P* < 0.05]. The maximum ACh-induced relaxation was further significantly different comparing H/R and control (*P* < 0.05; Brunner test). **(C)** COX inhibition [with indomethacin (Indo), EC_50_: 1.63^∗^10^-7^ mol/l (control) vs. 4.00^∗^10^-7^ mol/l (H/R); *P* < 0.05]. **(D)** K_Ca_3.1 inhibition [with TRAM34, EC_50_: 2.02^∗^10^-7^ mol/l (control) vs. 4.05^∗^10^-7^ mol/l (H/R): *P* < 0.05] and K_Ca_2.1 inhibition [with UCL 1684, EC_50_: 3.52^∗^10^-7^ mol/l (control) vs. 2.07^∗^10^-6^ mol/l (H/R): *P* < 0.05]. Mean ± SEM. (^∗^) indicates significant differences. **(E)** Relative x-fold mRNA expression of nNOS, iNOS and eNOS normalized to 18SrRNA under H/R relative to control conditions. H/R significantly decreased iNOS and eNOS mRNA expression levels. The average mRNA expression level under control conditions was arbitrarily given a value of 1 (2^0^) (^∗^*P* < 0.05; compared to control; Mann–Whitney Test; *n* = 8).

To simulate the effect of H/R in the kidney, hypoxia for 60 min and reoxygenation for 10 min were applied to isolated ILA from rats. Initial experiments excluded adverse effects of carbogen exposure compared to ambient pO_2_ on vasoreactivity of ILA (EC_50_: 3.3^∗^10^-8^ mol/l (carbogen) vs. 1.7^∗^10^-8^ mol/l (21% O_2_), **Figure 2A**).

In order to approach the *in vivo* situation, a non-constrictor concentration of NE (10^-9^ mol/l) was applied during the hypoxic period and the respective control period. Hypoxia in presence of NE followed by reoxygenation decreased the sensitivity to ACh compared to control conditions [EC_50_: 2.4^∗^10^-7^ mol/l (control) vs. 4.8^∗^10^-7^ mol/l (H/R); *P* = 0.004, **Figure 2A**]. Hypoxia without NE treatment did not affect the relaxation to ACh and NE did not affect the ACh-induced relaxation in the control group (data not shown).

### Endothelial-Dependent Dilatory Systems Are Not Responsible for the H/R Effect

To investigate the mechanisms behind the observed diminished vasodilatory response to H/R (hereafter: “H/R effect”), inhibitors of main endothelium-dependent dilatory systems were applied during the experiment. If any of the investigated systems is responsible for the H/R effect, the respective inhibition should align the concentration–response-relationship of control and H/R.

Inhibition of NO synthases (NOS) with L-NAME (10^-4^ mol/l) did not reverse the H/R effect [EC_50_: 3.3^∗^10^-7^ mol/l (control) vs. 9.5^∗^10^-7^ mol/l (H/R); *P* = 0.004]. However, it significantly reduced the maximum ACh effect [relaxation compared to PE contraction: 62.3 ± 4.9% (control) vs. 36.2 ± 5.2% (H/R); *P* = 0.007 **Figure 2B**]. The COX inhibitor indomethacin (10^-5^ mol/l) did not align the concentration–response-relationships of control and H/R [EC_50_: 1.6^∗^10^-7^ mol/l (control) vs. 4.0^∗^10^-7^ mol/l (H/R); *P* = 0.005, **Figure 2C**]. Further, neither K_Ca_3.1 inhibition by TRAM34 [10^-6^mol/l, EC_50_: 2.0^∗^10^-7^ mol/l (control) vs. 4.1^∗^10^-7^ mol/l (H/R); *P* = 0.005] nor K_Ca_2.1 inhibition by UCL1684 [10^-7^mol/l, EC_50_: 3.5^∗^10^-7^ mol/l (control) vs. 2.1^∗^10^-6^ mol/l (H/R); *P* = 0.03] eliminated the inhibitory effect of H/R on ACh-mediated relaxation (**Figure 2D**).

H/R treatment resulted in a significant downregulation of inducible NOS mRNA (0.45-fold; *P* = 0.025) and endothelial NOS mRNA (0.34-fold; *P* = 0.026) in rat ILA (**Figure 2E**).

### Impaired Vascular Smooth Muscle Cell Function in Rat Interlobar Arteries After H/R

**FIGURE 3:**
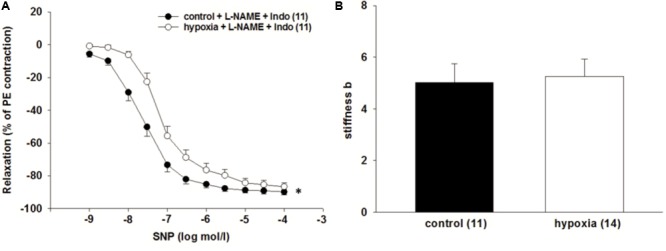
Effect of H/R on endothelium independent relaxation in isolated rat renal ILA. **(A)** H/R and simultaneous inhibition of the endothelial dilatory systems did not reverse the effect of H/R on ILA relaxation as indicated by significantly different EC_50_ [3.32^∗^10^-8^ mol/l (control) vs. 9.37^∗^10^-8^ mol/l (H/R); *P* < 0.05]. **(B)** Vascular wall stiffness parameter b (beta) did not significantly differ between both groups. Mean ± SEM. (^∗^) indicates significant differences.

After excluding that endothelial-dependent vasodilatory systems are responsible for the observed H/R effect, the endothelium-independent relaxation of ILA was investigated by application of the NO donor SNP and simultaneous inhibition of endothelial-dependent dilatory systems. H/R reduced the sensitivity of ILA to SNP during simultaneous inhibition of NOS (L-NAME, 10^-4^ mol/l) and COX (indomethacin, 10^-5^ mol/l), compared to control condition [EC_50_: 3.3^∗^10^-8^ mol/l (control) vs. 9.4^∗^10^-8^ mol/l (H/R); *P* = 0.01, **Figure 3A**], indicating that the H/R effect is independent of NO-bioavailability, but rather depends on an impaired VSMC function. Analysis of the vascular wall stiffness in non-stimulated vessels did not show significant differences between H/R treated and control vessels (**Figure 3B**).

### Superoxide Does Not Contribute to the H/R Effect

**FIGURE 4:**
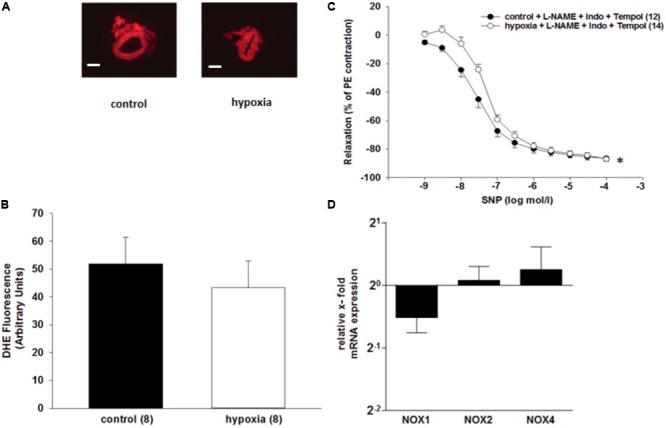
Analysis of superoxide in renal rat ILA. **(A)** Representative images of vascular rings loaded with DHE under control conditions and after H/R treatment (DHE concentration: 2^∗^10^-5^ mol/l, magnification: 200×; scale bar = 400 μm) **(B)** Analysis of DHE fluorescence showed no significant differences between both groups. **(C)** H/R and simultaneous inhibition of superoxide, using the superoxide dismutase mimetic tempol, did not reverse the H/R effect on endothelium-independent relaxation in isolated rat renal ILA precontracted with PE. [EC_50_: 3.34^∗^10^-8^ mol/l (control) vs. 6.75^∗^10^-8^ mol/l (H/R); *P* < 0.05]. **(D)** Relative x-fold mRNA expression of NADPH oxidase enzymes (NOX) normalized to 18SrRNA after H/R relative to control conditions. The analyzed NOX were not differentially regulated following H/R. The average mRNA expression level under control conditions was arbitrarily given a value of 1 (2^0^) (^∗^*P* < 0.05; compared to control; Mann–Whitney Test; *n* = 8). Mean ± SEM. (^∗^) indicates significant differences.

It was tested whether superoxide contributes to the impaired relaxation of ILA after H/R. The superoxide dismutase mimetic 4-hydroxy-TEMPO (tempol) did not influence the impaired relaxation to SNP during inhibition of the endothelium by L-NAME (10^-4^ mol/l) and indomethacin [10^-5^ mol/l, EC_50_: 3.3^∗^10^-8^ mol/l (control) vs. 6.8^∗^10^-8^ mol/l (H/R); *P* = 0.033, **Figure 4C**]. Moreover, superoxide concentration measured by DHE fluorescence did not significantly differ between ILA slices from H/R and control conditions and NADPH oxidase enzymes NOX1, NOX2, NOX4 were not differentially expressed following H/R, further indicating no major role of oxidative stress after H/R under these experimental conditions (**Figures 4A,B,D**).

### H/R Attenuates the Response to PDE5-Inhibition and Significantly Decreases cGMP Concentration

**FIGURE 5:**
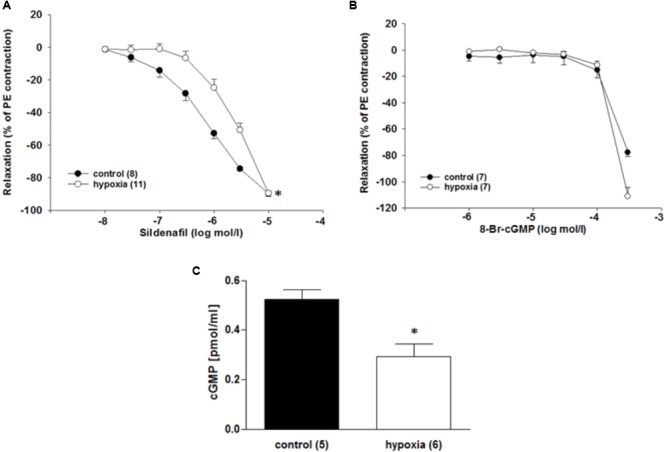
Effect of H/R on the cGMP pathway in rat renal ILA. **(A)** The H/R effect on ILA relaxation was not abolished by PDE5 inhibition using sildenafil as indicated by significant differences in the EC_50_ [1.18^∗^10^-6^ mol/l (control) vs. 7.14^∗^10^-6^ mol/l (H/R); *P* < 0.05]. **(B)** The cGMP analog 8-Br-cGMP reversed the H/R effect and abolished significant differences between the two groups. **(C)** H/R significantly decreased cGMP concentration in ILA (*P* < 0.05). Mean ± SEM. (^∗^) indicates significant differences.

By using the PDE5 inhibitor sildenafil and the cell-permeable cGMP analog 8-bromoguanosine 3’,5’-cyclic monophosphate sodium salt (8-Br-cGMP), it was investigated whether the function of key enzymes in cGMP pathway changes under H/R. The relaxation to the PDE5 inhibitor sildenafil (10^-5^ mol/l) was reduced in ILA after H/R compared to control conditions [EC_50_: 1.2^∗^10^-6^ mol/l (control) vs. 7.1^∗^10^-6^ mol/l (H/R); *P* = 0.001, **Figure 5A**]. 8-Br-cGMP (10^-6^ to 3^∗^10^-4^ mol/l) relaxed ILA similarly under H/R and control conditions (**Figure 5B**).

The cGMP concentrations were lower after H/R compared to the control conditions [0.52 ± 0.04 pmol/ml (control) vs. 0.29 ± 0.05 pmol/ml (H/R); *P* = 0.017; **Figure 5C**]. Moreover, PDE5 mRNA is highly expressed in ILA, but not differentially regulated after H/R rather pointing to a dysregulation of cGMP production (x-fold mRNA expression H/R relative to control: 1.16 ± 0.12; *P* = 0.396).

**FIGURE 6:**
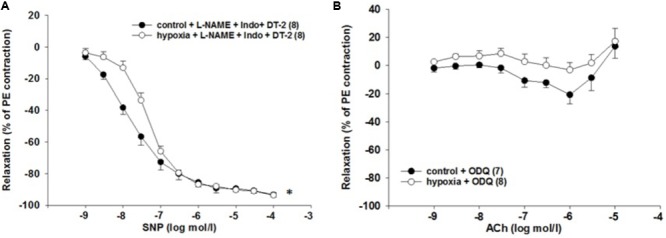
Effect of H/R on sGC-dependent relaxation in isolated rat renal interlobar arteries (ILAs) precontracted with PE. **(A)** Inhibition of PKG kinase by DT-2 could not reverse H/R effect on ILA relaxation [EC_50:_ 2.14^∗^10^-8^ mol/l (control) vs. 4.88^∗^10^-8^ mol/l (H/R); *P* < 0.05]. Mean ± SEM. (^∗^) indicates significant differences. **(B)** The sGC inhibitor ODQ abolished the H/R effect on ILA relaxation and aligned control and H/R dose–response relationships [8.90^∗^10^-7^ mol/l (control) vs. 1.70^∗^10^-6^ mol/l (H/R); EC_50_ not significant, ANOVA not significant].

We also tested the role of PKG, which is involved in the NO signaling. SNP was applied while the endothelial dilator function was inhibited by L-NAME (10^-4^ mol/l) and indomethacin (10^-5^ mol/l). PKG inhibition by DT-2 (10^-7^ mol/l) did not affect the impaired relaxation due to H/R [EC_50_: 2.1^∗^10^-8^ mol/l (control) vs. 4.9^∗^10^-8^ mol/l (H/R); *P* = 0.013, **Figure 6A**].

### sGC Inhibition Reverses the H/R Effect on ILA Relaxation

Activation of the sGC enzyme via NO results in cGMP production and vessel relaxation. Therefore, the observed reduced cGMP levels leading to impaired relaxation might be caused by sGC dysfunction. The sensitivity to ACh was similar comparing H/R and control under ODQ treatment [EC_50_: 8.9^∗^10^-7^ mol/l (control) vs. 1.7^∗^10^-6^ mol/l (H/R), *p* = 0.281]. Thus, inhibition of sGC using the inhibitor ODQ reverses the initially observed H/R effect on relaxation and aligns the concentration–response relationships of control and H/R treatment (**Figure 6B**). **Table 2** summarizes the EC_50_ and the respective maximum response of the presented small vessel myograph experiments.

**Table 2:** EC_50_ (mean ± SEM) and maximum response (mean ± SEM) of small vessel myograph studies; # indicates *P* < 0.05.

Treatment	EC_50_ control (mol/l)	EC_50_ hypoxia (mol/l)	Maximum response control (%)	Maximum response hypoxia (%)
ACh-CRC	2.37^∗^10^-7^± 3.32^∗^10^-8^	4.75^∗^10^-7^± 6.29+10^-8^ #	–82.79 ± 3.78	–75.04 ± 2.77
L-NAME+ACh-CRC	3.33^∗^10^-7^± 4.99^∗^10^-8^	9.54^∗^10^-7^± 2.43^∗^10^-7^ #	–62.32 ± 4.86	–36.22 ± 5.22 #
Indo+ACh-CRC	1.63^∗^10^-7^± 3.23^∗^10^-8^	4.00^∗^10^-7^± 6.43^∗^10^-8^ #	–87.78 ± 1.92	–82.06 ± 2.45
TRAM34+ACh-CRC	2.02^∗^10^-7^± 6.08^∗^10^-8^	4.05^∗^10^-7^± 8.00^∗^10^-8^ #	–83.66 ± 1.75	–81.43 ± 2.15
UCL1684+ACh-CRC	3.52^∗^10^-7^± 6.41^∗^10^-8^	2.07^∗^10^-6^± 1.36^∗^10^-6^ #	–87.54 ± 4.51	–76.78 ± 2.77 #
L-NAME+Indo+SNP-CRC	3.32^∗^10^-8^± 1.00^∗^10^-8^	9.37^∗^10^-8^± 2.70^∗^10^-8^ #	–89.77 ± 1.82	–86.63 ± 2.45
L-NAME+Indo+Tempol+SNP-CRC	3.34^∗^10^-8^± 6.58^∗^10^-9^	6.75^∗^10^-8^± 1.53^∗^10^-8^ #	–86.53 ± 2.02	–87.02 ± 1.81
Sildenafil-CRC	1.18^∗^10^-6^± 3.00^∗^10^-7^	7.14^∗^10^-6^± 2.51^∗^10^-6^ #	–89.69 ± 1.91	–89.55 ± 1.30
L-NAME+Indo+DT-2+SNP-CRC	2.14^∗^10^-8^± 5.45^∗^10^-9^	4.88^∗^10^-8^± 7.91^∗^10^-9^ #	–93.37 ± 1.50	–93.64 ± 1.78
ODQ+ACh-CRC	8.90^∗^10^-7^± 5.34^∗^10^-7^	1.70^∗^10^-6^± 5.68^∗^10^-7^	–20.76 ± 6.64	–3.03 ± 5.10

## Discussion

This study shows that the impaired relaxation of rat ILA to ACh after treatment with H/R, in the presence of physiological level of NE, is mediated by modified signaling in VSMCs. No functional impairment of the endothelial dilatory NOS- and COX-systems as well as EDHF was detected under these experimental conditions. Further, PDE5 and PKG, the downstream target of cGMP, seem not to be responsible for the H/R effect. Noticeably, reduced cGMP concentrations were found after H/R. We demonstrate here that sGC dysfunction caused by H/R results in impaired relaxation of ILA, which appears independent of NO-bioavailability.

Altered renal perfusion is considered to play a key role in the pathophysiological events following IRI. Renal vessels are effectors as well as targets in the pathophysiological course of ischemia and the subsequent H/R stage. The observation of increased vasoreactivity of mouse ILA to Ang II after H/R supports the assumption that enhanced vessel tone contributes to IRI (Kaufmann et al., 2015; Braun et al., 2017; Pahlitzsch et al., 2018). Since vascular tone results from the balance of constrictor and dilator systems, it was hypothesized that increased vasoreactivity in H/R may also result from impaired vasodilator systems, which can mediate relaxation via endothelial-dependent or -independent mechanisms. NO is an important dilatory factor in the kidney and contributes to the ACh-induced renal relaxation (Tai et al., 2004; Patzak and Persson, 2007). Enzymatic blockade of NO synthases by L-NAME did not prevent the reduced relaxation after H/R compared to control but decreased the sensitivity to ACh similarly in both groups. The maximum relaxation was smaller in H/R compared to control. These results suggest no role of the endothelial NO-system for reduced relaxation in H/R. However, our functional data point to an enhanced NO-bioavailability at higher ACh concentrations after H/R. As reported in recent studies, increased NO-bioavailability could be the result of an increased NO production from nitrite under hypoxic conditions (Feelisch et al., 2008; Liu et al., 2015). Interestingly, H/R significantly decreased endothelial NOS and inducible NOS expression at the transcriptional level, but this result is limited by the lack of protein expression and activity data. A time-dependent differential mRNA regulation of NOS enzymes cannot be excluded.

Indomethacin, a COX pathways inhibitor, neither affected the relaxation to ACh of ILA, nor influenced the difference in the ACh response between H/R and control. This observation is in line with findings in isolated rat renal arteries and isolated perfused rat afferent arterioles (Wang et al., 2003; Grbovic et al., 2008). In contrast, experiments in anesthetized rats revealed a 20–40% contribution of the indomethacin inhibited systems to the control of renal perfusion (Dautzenberg and Just, 2013). These contrary results may reflect a different function of indomethacin inhibitable systems in the *in vivo* vs. *in vitro* situation as well as different expression of COX in larger arteries vs. arterioles.

Endothelium-derived hyperpolarization has been identified as a factor for renal vessel relaxation (Edgley et al., 2008; Michel et al., 2008; Simonet et al., 2012; Dautzenberg and Just, 2013; Salomonsson et al., 2017). The endothelial hyperpolarization, which is mediated by Ca^2+^-activated K^+^-channels (K_Ca_) initializes the EDHF-dilator response. Two K_Ca_ subtypes, namely the intermediate-conductance K_Ca_ (K_Ca3.1_) and the small-conductance K_Ca_ type 2 (K_Ca2.1_) contribute mainly to the hyperpolarization (Grgic et al., 2009). Inhibition of K_Ca3.1_ and K_Ca2.1_ with TRAM34 and UCL 1684, respectively, did not exert significant effects on the ACh concentration–response relationships. Most importantly the differences in ACh sensitivity between the H/R and control group remained, suggesting no significant role of EDHF for the H/R effect. In summary, endothelial-dependent vasodilatory systems are not responsible for diminished relaxation following H/R.

The H/R-induced impairment of relaxation only occurs in vessels treated with low concentration of NE (10^-9^ mol/l) during the hypoxic phase. This concentration is within the range of physiological NE plasma concentrations (Kawada et al., 2014) and did not affect the tone or relaxation to ACh in control vessels. It has been shown that H/R in combination with NE (10^-9^ mol/l) enhanced constriction to Ang II in mouse ILA (Kaufmann et al., 2015). The mechanisms of the combined effect of activation of the sympathetic system and H/R are not well understood.

An incomplete relaxation of rat arterioles in response to the removal of NE treatment (10^-5.5^ mol/l) has been reported (Martinez-Lemus et al., 2004). The incomplete relaxation was interpreted as a mechanoadaptive mechanism of VSMCs to the NE treatment. However, structural changes of ILA following H/R do not appear to be important in the present study. Vessels did not differ regarding vessel stiffness calculated by the parameter beta comparing control and H/R group, indicating similar passive properties.

Previous studies reported enhanced oxidative stress following H/R, which potentiates IRI damage to the kidney and increases vasoconstriction (Jankauskas et al., 2016; Pahlitzsch et al., 2018). To test if oxidative stress contributed to H/R-mediated vascular dysfunction in the present study, DHE fluorescence was assessed. However, no difference in fluorescence was observed when comparing H/R-treated and control vessels. Furthermore, tempol, a superoxide dismutase mimetic, did not affect the difference in the SNP sensitivity between vessels after H/R and control treatment. NADPH oxidase enzymes were not differentially regulated. Taken together, our data do not indicate a major role of superoxide in this context. However, the involvement of reactive oxygen species other than superoxide cannot be excluded (Huang et al., 2016).

Treatment with the NO donor SNP dilated control vessels in a concentration-dependent manner, and this response was reduced in H/R-treated vessels. This observation locate the mechanisms, by which H/R diminishes relaxation to ACh, in the VSMCs. The main receptor for NO in VSMCs is sGC, which catalyzes the production of cGMP. The latter activates PKG by several cellular mechanisms leading to reduced cytosolic calcium concentration and/or calcium sensitivity of contraction and subsequently to vasodilation. The cGMP concentration results from its production as well as from its degradation by phosphodiesterases (PDE). PDE5 is strongly expressed in ILA and may significantly contribute to the regulation of the cGMP level. Inhibition of PDE5 by sildenafil induced relaxation of vessels in the present study. The sensitivity to sildenafil was reduced after H/R compared to control. To clarify if this reduced sensitivity is due to (i) lower cGMP production by sGC, (ii) changes in PDE5 expression, or (iii) downstream signaling, further experiments were performed. The stable cGMP analog 8-Br-cGMP relaxed control and H/R-treated ILA in a similar way and, in line with previous studies, only at high concentrations (Goulopoulou et al., 2015). Inhibition of PKG, an important kinase downstream of cGMP, did not abolish the differences in SNP responses between H/R and controls further locating the H/R effect to other up- or downstream targets. Moreover, cGMP concentrations were significantly decreased following H/R pointing to disturbed or lower cGMP production by sGC. Therefore, we investigated the effects of sGC inhibition on ILA relaxation using its heme-site inhibitor ODQ. Inhibition of sGC signaling reversed the H/R effect on ILA relaxation pointing to a disturbed sGC signaling. Our results suggest that H/R adversely affects sGC function leading to impaired relaxation in rat ILA.

Due to our *ex vivo* model of isolated vessels, limitations occur in our study, which might influence the interpretation of our results. In contrast to the *in vivo* situation, our vessels are not exposed to a systemic or local renin-angiotensin system (RAS) or secreted substances from surrounding or adjacent cells, which might essentially influence vessel tone. NE was applied to simulate sympathetic activity, as it was found to be crucial for the reproduction of an H/R effect. Currently, there is no adequate model available that offers accessibility of small vessels and at the same time, closely simulates the complex *in vivo* interplay of RAS, sympathetic system, vasoactive substances and vasoreactivity. Therefore, it should be acknowledged that the conclusion reached here refers to isolated vessels and might not be directly transferable to *in vivo* situation.

Moreover, this study is restricted to investigations of the sGC/cGMP pathway and its role in the H/R effect of impaired relaxation. While we could show that sGC dysfunction following H/R leads to diminished relaxation of interlobar arteries, we did not analyze a possible role of adenylatecyclase/cAMP signaling in our experimental setup. It is imaginable that decreased cAMP levels and/or a dysfunction of adenylatecyclase might contribute to the H/R effect. This assumption warrant further investigations.

## Conclusion

H/R impairs ACh-induced relaxation by an endothelium-independent way in vascular smooth muscles cells. The mechanism whereby H/R induced impaired relaxation may include sGC dysfunction and subsequently decreased cGMP production. The impaired relaxation due to H/R may be a pathogenetic factor for reduced renal perfusion and delayed reperfusion in ischemia reperfusion injury.

## Author Contributions

All authors have seen and approved the final version of the manuscript. DB, CZ, ML, and SD performed most of the experiments with arteries and arterial tissue, respectively, the statistical analysis, and contributed intellectually to the writing of the manuscript. SG and HS designed primers, performed qPCR, analyzed and interpreted data, and revised the article for important intellectual content. RS, ML, PP, MC, and AP made substantial contribution to the conception of the article, the data interpretation, drafting of the manuscript, and revised the article for important intellectual content.

## Conflict of Interest Statement

The authors SG and HS were employed by Bayer Pharma AG, Wuppertal, Germany. The other authors declare that the research was conducted in the absence of any commercial or financial relationships that could be construed as a potential conflict of interest.

## Footnotes

1 https://www.r-project.org/
